# Incidence and mortality of tuberculosis before and after initiation of antiretroviral therapy: an HIV cohort study in India

**DOI:** 10.7448/IAS.17.1.19251

**Published:** 2014-12-09

**Authors:** Gerardo Alvarez-Uria, Raghavakalyan Pakam, Manoranjan Midde, Praveen K Naik

**Affiliations:** Department of Infectious Diseases, Rural Development Trust Hospital, Anantapur, Andhra Pradesh, India

**Keywords:** HIV, tuberculosis, mortality, incidence, India, rural, antiretroviral therapy, gender, CD4 lymphocyte count, risk

## Abstract

**Introduction:**

India has the highest burden of tuberculosis (TB) in the world, but the epidemiology of HIV-associated TB is not well known.

**Methods:**

We describe the incidence and the mortality of TB from HIV diagnosis to antiretroviral therapy (ART) initiation (pre-ART group) and after ART initiation (on-ART group) in an HIV cohort study in Anantapur, India. Multivariable analysis of factors associated with TB was performed using competing risk regression and restricted cubic spline methods.

**Results:**

A total of 4590 patients and 3133 person-years (py) of follow-up were included in the pre-ART group, and 3784 patients and 4756 py were included in the on-ART group. In the pre-ART group, the incidence of TB was high during the first month after HIV diagnosis and dropped nearly four times soon after. In the on-ART group, the incidence of TB increased after ART initiation reaching a peak in the third month. The probability of having TB within 30 months was 22.3% (95% confidence interval [CI], 21.1–23.6) in the pre-ART group and 17.8% (95% CI, 16.3–19.3) in the on-ART group. In a multivariable analysis, women had a lower risk of TB in both groups. Poor socio-economical conditions were associated with an increased risk of TB in the pre-ART group, but not in the group on-ART. While the association between low CD4 counts and TB was strong in the pre-ART group, this association was weaker in the on-ART group, and the highest risk of TB was seen in those patients with CD4 counts around 110 cells/mm^3^. The cumulative incidence of mortality at 12 months in patients with TB was 29.6% (95% CI, 26.9–32.6) in pre-ART TB and 34.9% (95% CI, 31–39.1) in on-ART TB. Half deaths before ART initiation and two thirds of deaths after ART initiation occurred in patients with TB.

**Conclusions:**

The high incidence and mortality of TB seen in this study underscore the urgent need to improve the prevention and diagnosis of HIV-associated TB in India. We found substantial differences between TB before and after ART initiation.

## Introduction

Tuberculosis (TB) is the leading cause of death among HIV-positive people living in low- and middle-income countries [1]. The rollout of free antiretroviral therapy (ART) in these countries is likely to have a major impact in the epidemiology of HIV-associated TB. In areas of endemic TB, the use of ART is associated with a significant reduction in the incidence of TB [2]. On the other hand, patients who initiate ART are at higher risk of experiencing TB during the first months of treatment [3,4]. However, differences between TB before ART and TB after ART initiation are not well known [5].

In India, 5.9% of the 2,200,000 incident cases and 15.6% of the 270,000 deaths due to TB occur in HIV-positive patients, and the estimated case fatality rate of HIV-positive patients with TB is 32.3% [6]. Despite the high burden of TB in India, there is scant information about the epidemiology of HIV-related TB. It is estimated that 41% of TB cases are not diagnosed or are not notified to the national TB programme, and only 59% of TB cases are HIV tested [6]. Although some cross-sectional studies have reported 17 to 30% prevalence of TB among HIV-positive patients [7–9], there is a paucity of data about the incidence of TB after the implementation of free ART by the Government of India [10]. The aim of this study was to describe the epidemiology of TB in a large cohort of HIV-positive patients. In particular, we aimed to investigate differences in TB before and after initiation of ART.

## Methods

### Setting

The study was performed in Anantapur, Andhra Pradesh, India. In Anantapur, 72% of the population live in rural areas [11]. The HIV epidemic is largely driven by heterosexual transmission, and it is characterized by low CD4 cell counts at HIV presentation, poor socio-economic conditions and high levels of illiteracy [12–14].

The Vicente Ferrer HIV Cohort Study (VFHCS) is an open cohort study of HIV-positive patients who have attended Bathalapalli Rural Development Trust (RDT) Hospital. This hospital belongs to a non-governmental organization, and provides medical care to HIV-positive people free of charge, including ART and anti-TB therapy. The cohort is fairly representative of the population diagnosed with HIV in the district, as it covers approximately 70% of all HIV-positive people registered in the district [15]. The baseline characteristics of the cohort have been described in detail elsewhere [12].

To study the incidence of TB from HIV diagnosis to ART initiation, we selected adults (age >16 years) from the VFHCS database living in Anantapur and diagnosed with HIV from 1 January 2010 to 1 February 2013 (pre-ART group). To study the incidence of TB after ART initiation, we selected adults (age >16 years) from the VFHCS database living in Anantapur who initiated ART from 1 January 2010 to 1 February 2013 (on-ART group). Patients included in the pre-ART group who experienced pre-ART TB and started ART during the study period were also included in the on-ART group and were considered at risk of on-ART TB.

### Clinical management and diagnosis of TB

According to World Health Organization (WHO) recommendations for the definition of TB case and the locally available standard of care [16], the diagnosis of TB was made based on the presence of acid-fast bacilli on sputum smear, caseating or necrotizing granuloma in clinical specimens, or clinical presentation suggestive of TB along with supportive findings in the chest radiograph, abdominal ultrasound or laboratory results from biological fluids. Acid-fast bacilli staining of sputum and chest radiograph were performed for all patients with a clinical suspicion of TB. Analysis of cerebrospinal fluid, pleural fluid or ascitic fluid was performed if there were signs of neurological involvement, pleural fluid in the chest radiograph or ascites, respectively. In smear-negative patients complaining of important weight loss, an abdominal ultrasound was performed to search for signs of abdominal TB [17,18].

Patients with TB not on ART were counselled to start ART after two weeks of anti-TB therapy. In patients without TB, ART was initiated by clinical criteria (clinical stage three or four of the WHO) or by immunological criteria (CD4 count <250 cells/mm^3^ before November 4th 2011, and <350 cells/mm^3^ thereafter) according to the Indian National Guidelines [19,20]. During the study period, isoniazid preventive therapy was not provided, but co-trimoxazole preventive therapy was given to all patients with TB.

### Definitions

Designation of the community of patients was performed by self-identification. Scheduled caste (SC) community is marginalized in the traditional Hindu caste hierarchy and, therefore, suffers social and economic exclusion and disadvantage. Scheduled tribe (ST) community is generally geographically isolated with limited economic and social contact with the rest of the population. Backward castes (BC) form a collection of “intermediate” castes considered low in the traditional caste hierarchy, but above SCs [21]. SC, ST and BC communities are considered socially disadvantaged communities and are supported by positive discrimination schemes operated by the Government of India. Patients not belonging to SC, ST or BC communities were included in the other castes (OC) group.

### Statistical analysis

Statistical analysis was performed using the Stata Statistical Software (Stata Corporation. Release 12.1; College Station, TX). Time-to-event methods were used. In the pre-ART group, time was measured from HIV diagnosis to the first episode of TB, ART initiation or death, whatever occurred first. In the on-ART group, time was measured from ART initiation to the first episode of TB or death, whatever occurred first. Only one episode of TB per patient was allowed in each group. Patients who did not experience any of the mentioned events were censored at February 1st 2013 (end of the study period).

To estimate the cumulative incidence and the multivariable analyses of factors associated with the event of interest (TB or mortality), we followed two different strategies according to the presence or absence of competing events:

1. When there were not competing events, we used Kaplan-Meier and Cox proportional hazard regression. These methods were used in the analysis of mortality of pre-ART TB and on-ART TB.
2. When there were competing events, Kaplan-Meier and Cox proportional hazard models were not used because of violation of the assumption that the distribution of censoring times and the time to event distribution are independent of each other. For example, when studying the cumulative incidence or factors associated with TB before ART initiation, a group of patients will be censored at death or at the date of ART initiation despite the fact that patients who died or started ART will not be able to experience pre-ART TB. In Cox regression models or Kaplan-Meier estimates these patients are considered at risk of pre-ART TB, leading to an overestimation of the event of interest (such as pre-ART TB in this example) and inaccurate estimation of hazard ratios in multivariable analysis [22,23]. Thus, in the presence of competing events, cumulative incidences and multivariable analysis were estimated using competing risk methods [24,25]. Competing risk regression models estimate sub-distribution hazard ratios, which can be interpreted similarly to hazard ratios estimated by Cox proportional hazard models, but they take into account the hazard of competing events.

Hazard densities were estimated using Epanechnikov kernel functions [26].

To relax the linearity assumption in regression models and to allow for a flexible representation of the relationship between continuous covariates and the event of interest, continuous variables were transformed using restricted cubic splines with five knots [27]. The relationship of continuous covariates with the event of interest was presented graphically [28]. To facilitate the interpretation of these graphs, the selection of the reference value was performed with intention of showing hazard ratios above one. To facilitate comparisons, we used the same y-axis scale and similar reference values in both groups. CD4 lymphocyte count was modelled as a time-dependent predictor.

The VFHCS was performed according to the principles of the Declaration of Helsinki, and was approved by the Ethics Committee of the RDT Hospital. Written informed consent was given by patients or caretakers for their information to be stored in the study database and used for research.

## Results

### Baseline characteristics

During the study period, we identified 4590 patients diagnosed with HIV (pre-ART group), and 3784 patients who started ART (on-ART group). The median baseline age was 34.9 years (interquartile range [IQR] 28–40.2) in the pre-ART group, and 32.2 years (IQR 28–40) in the on-ART group. The median baseline CD4 lymphocyte count was 209 cells/mm^3^ (IQR 102–393) in the pre-ART group, and 190 cells/mm^3^ (IQR 111–267) in the on-ART group. Other socio-demographic characteristics are described in Table 1.

**Table 1:** Baseline characteristics and multivariable analysis of socio-demographic risk factors associated with TB adjusted by age and time-updated CD4 lymphocyte counts

	Baseline characteristics		TB incidence		Factors associated with TB	
	
	Pre-ART	On-ART	Pre-ART	On-ART	Pre-ART	On-ART
	*N* (%)	*N* (%)	IR/100 py	IR/100 py	as-HR (95% CI)	as-HR (95% CI)
Gender						
Male	2493 (54.31)	1882 (49.74)	40 (37–43.2)	16.5 (14.9–18.3)	1 (Reference)	1 (Reference)
Female	2097 (45.69)	1902 (50.26)	22.9 (20.6–25.4)	6.7 (5.8–7.8)	0.70* (0.61–0.80)	0.52* (0.42–0.64)
Community						
OC	940 (20.48)	867 (22.91)	27 (23.2–31.4)	11.4 (9.6–13.6)	1 (Reference)	1 (Reference)
BC	2265 (49.35)	1884 (49.79)	30.4 (27.8–33.3)	11.5 (10.2–13)	1.20* (1.01–1.44)	1.00 (0.80–1.24)
SC	1019 (22.2)	759 (20.06)	37.8 (33.5–42.7)	11.3 (9.4–13.7)	1.49* (1.22–1.83)	0.97 (0.74–1.26)
ST	366 (7.97)	274 (7.24)	34.7 (27.9–43)	10 (7.2–13.9)	1.39* (1.05–1.83)	0.99 (0.67–1.45)
Illiteracy						
No	2124 (46.27)	1800 (47.57)	25.6 (23.2–28.3)	11.8 (10.5–13.3)	1 (Reference)	1 (Reference)
Yes	2466 (53.73)	1984 (52.43)	37.4 (34.5–40.5)	11 (9.7–12.3)	1.29* (1.13–1.48)	1.06 (0.88–1.28)

* *p*<0.05. ART = antiretroviral therapy; as-HR = adjusted sub-hazard ratio; IR/100 py = incidence rate per 100 person-years; BC = backward castes; OC = other castes; SC = scheduled castes; ST = scheduled tribes; TB = tuberculosis. Overall *p*-values of community for TB risk were 0.0007 in the pre-ART group and 0.99 in the on-ART group.

### Risk factors associated with TB

In the pre-ART group, 993 patients experienced TB, 232 died and 2102 started ART during 3133 person-years (py) of follow-up. The median time from HIV diagnosis to TB was 0.2 months (IQR 0.07–0.69), from HIV diagnosis to death was 1.71 months (IQR 0.36–5.57), and from HIV diagnosis to ART initiation was 0.76 months (IQR 0.26–4.17).

In the on-ART group, 540 patients experienced TB, and 364 died during 4756 py of follow-up. The median time from ART initiation to TB was 2.8 months (IQR 0.7–7.9), and from ART initiation to death was 5.8 months (IQR 2–11.9).

**Figure 1:**
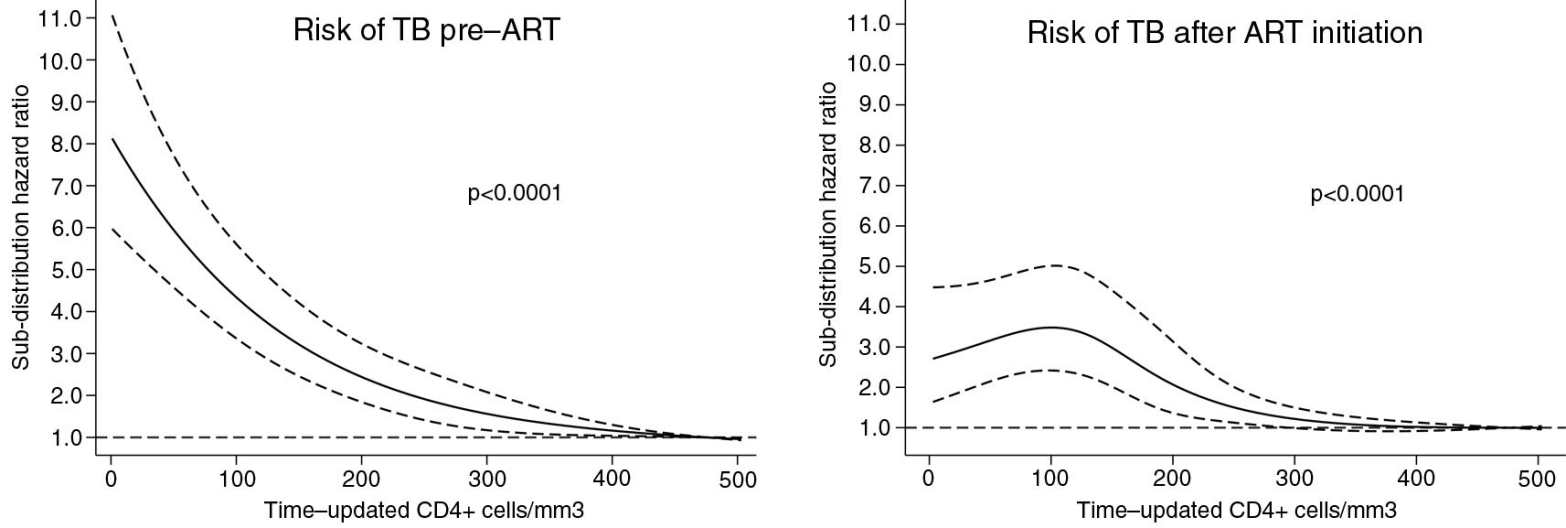
Risk of tuberculosis (TB) with 95% confidence intervals according to time-updated CD4+ lymphocyte counts from HIV diagnosis to antiretroviral therapy (ART) initiation and after ART initiation.

**Figure 2:**
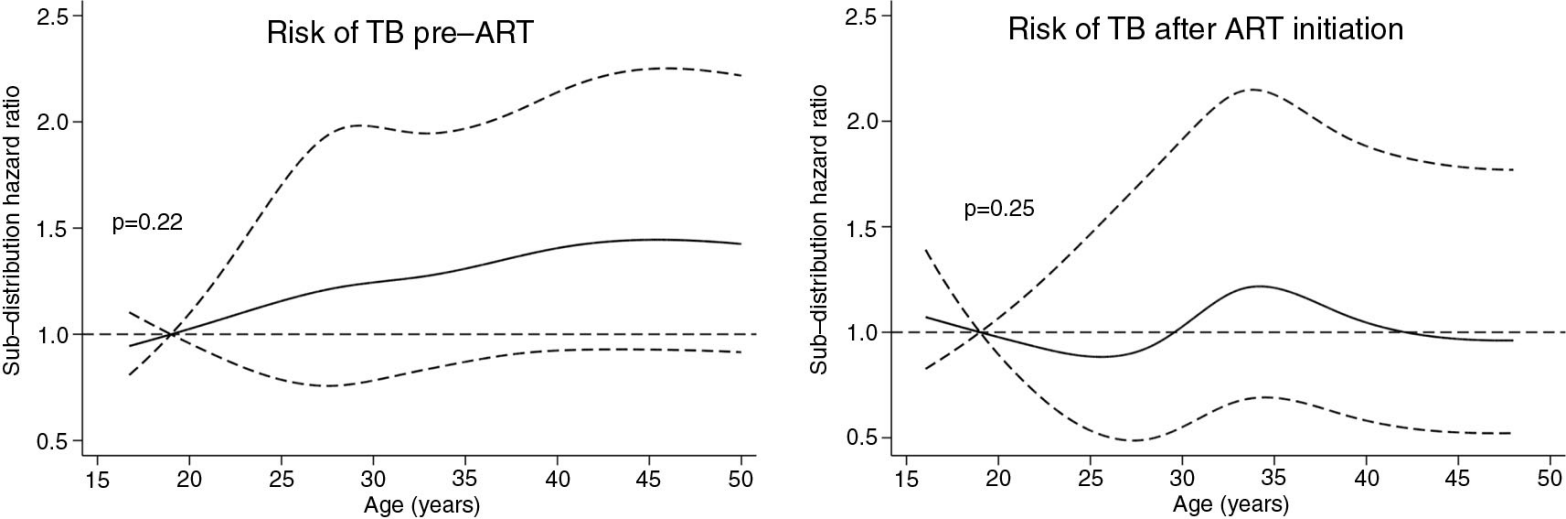
Risk of tuberculosis (TB) with 95% confidence intervals according to baseline age from HIV diagnosis to antiretroviral therapy (ART) initiation and after ART initiation.

We performed a multivariable analysis including gender, community, illiteracy and restricted cubic splines of baseline age and time-updated CD4 lymphocyte counts. We used competing risk regression with ART initiation (only in the pre-ART group) and death as competing events. The sub-distribution hazard ratios for TB of gender, communities and illiteracy are presented in Table 1. As the sub-distribution hazard ratios of restricted cubic splines for age and CD4 counts are not readily interpretable, these are presented graphically in Figures 1 and 2. Female gender was a protective factor for TB. Not belonging to OC community and illiteracy were associated with higher risk of TB only in the pre-ART group. The relation between risk of TB and CD4 lymphocyte counts is presented in Figure 1. While in the pre-ART group there was a strong association between low CD4 counts and TB, this association was weaker in the on-ART group, and the highest risk of TB was seen in those patients with CD4 counts around 110 cells/mm^3^. We did not find a significant association between age and TB (Figure 2).

### Incidence rates of TB

The incidence rate of pre-ART TB was 31.7/100 py (95% CI, 29.8–33.7). The incidence rate of on-ART TB was 11.4/100 py (95% CI, 10.4–12.4). The incidence rate ratio of pre-ART TB compared with on-ART TB was 2.8 (95% CI, 2.5–3.1). However, in patients who had a follow-up >six months without experiencing any of events of interest, the incidence rate ratio of TB was 0.78 (95% CI, 0.6–1.03).

The incidence rates of TB at different periods are described in Table 2. The pre-ART group had higher incidence rates of TB during the first three months, but lower thereafter. In both groups, the highest incidence rates were observed during the first three months of follow-up.

**Table 2:** Incidence rates of TB from HIV diagnosis to ART initiation and after ART initiation in Anantapur, India

Time period	Person-years	TB cases	Incidence rate (95% CI)
Since HIV diagnosis			
0–3 months	621.17	868	139.74 (130.74–149.35)
3–6 months	452.84	40	8.83 (6.48–12.04)
6–12 months	722.78	44	6.09 (4.53–8.18)
1–2 years	935.67	34	3.63 (2.6–5.09)
2–3 years	390.12	7	1.79 (0.86–3.76)
Since ART initiation			
0–3 months	854.2	281	32.9 (29.27–36.98)
3–6 months	730.05	90	12.33 (10.03–15.16)
6–12 months	1178.48	94	7.98 (6.52–9.76)
1–2 years	1421.24	58	4.08 (3.15–5.28)
2–3 years	569.77	17	2.98 (1.85–4.8)

CI=confidence interval; TB=tuberculosis; ART=antiretroviral therapy.

**Figure 3:**
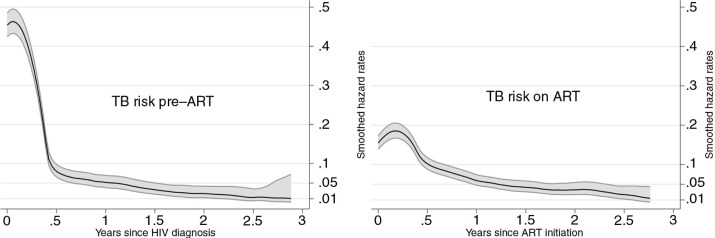
Kernel smoothed hazard of tuberculosis (TB) with 95% confidence intervals from HIV diagnosis to antiretroviral therapy (ART) initiation and after ART initiation.

Figure 3 describes the smoothed hazards of TB. In the pre-ART group, the hazard of TB was extremely high during the first month after HIV diagnosis and dropped nearly four times by the sixth month. Thereafter, a slow reduction in the hazard of TB was observed. In the on-ART group, the hazard of TB increased after ART initiation reaching a peak in the third month. Thereafter, we observed a rapid decline until the sixth month, and a slower reduction thenceforth.

### Cumulative incidence of TB

**Figure 4:**
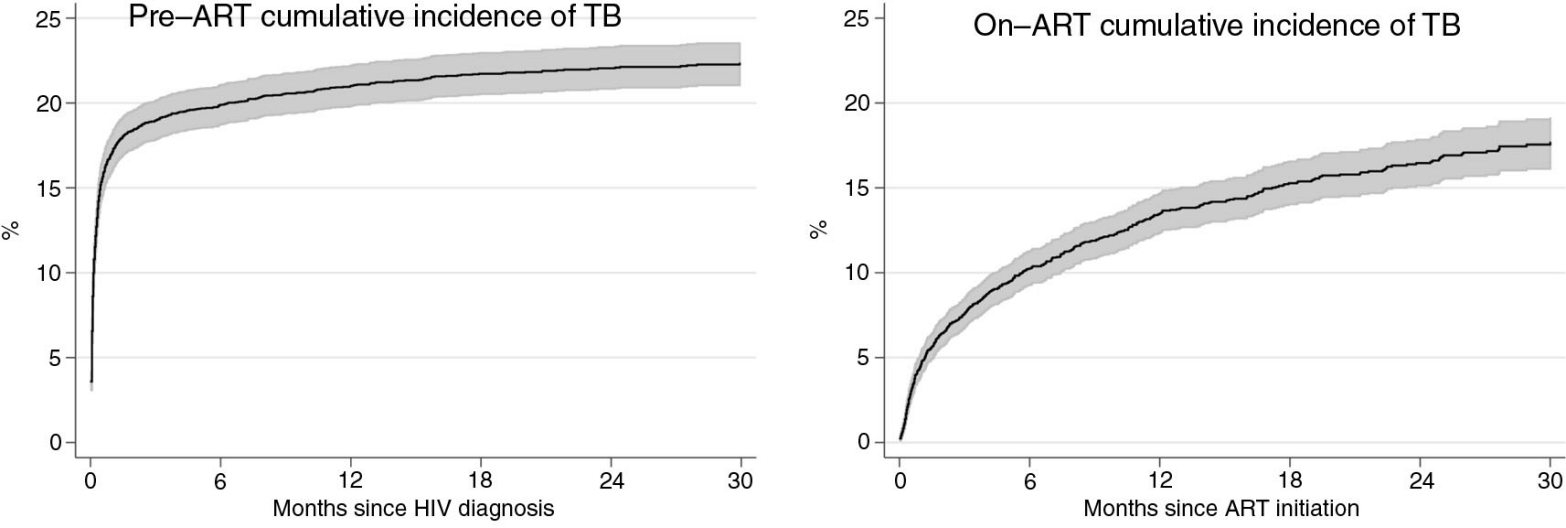
Cumulative incidence of tuberculosis (TB) with 95% confidence intervals from HIV diagnosis to antiretroviral therapy (ART) initiation and after ART initiation.

Figure 4 describes the cumulative incidence of TB adjusted for death as a competing risk. In the pre-ART group, ART initiation was also considered a competing risk, thus episodes TB that occurred after ART initiation were not included. The cumulative incidence of TB at 30 months was 22.3% (95% CI, 21.1–23.6) in the pre-ART group and 17.8% (95% CI, 16.3–19.3) in the on-ART group.

### Mortality in patients with TB

We studied the mortality in patients who experienced TB before ART initiation (pre-ART TB) and after ART initiation (on-ART TB). Patients were followed up from TB diagnosis to death. Patients who did not die were censored at the end of the study period. The mortality rate was 21.3/100 py (95% CI, 19.2–23.7) in patients with pre-ART TB and 26.1/100 py (95% CI, 22.9–29.8) in patients with on-ART TB. The mortality rates at different periods after the diagnosis of TB are described in Table 3. Patients diagnosed with TB after ART initiation had higher mortality rates during the first six months after TB diagnosis (incidence rate ratio 1.32, 95% CI 1.06–1.63). After six months since TB diagnosis, the mortality rates were similar in both groups.

**Table 3:** Mortality rates after TB diagnosis in Anantapur, India

Time period	Person-years	Deaths	Mortality rate (95% CI)
Pre-ART TB			
0–3 months	216.15	179	82.81 (71.53–95.88)
3–6 months	195.53	48	24.55 (18.5–32.57)
6–12 months	362.6	66	18.2 (14.3–23.17)
1–2 years	546.26	46	8.42 (6.31–11.24)
2–3 years	265.35	15	5.65 (3.41–9.38)
On-ART TB			
0–3 months	112.85	121	107.22 (89.72–128.13)
3–6 months	99.95	34	34.02 (24.31–47.61)
6–12 months	181.54	33	18.18 (12.92–25.57)
1–2 years	272.11	22	8.08 (5.32–12.28)
2–3 years	140.98	11	7.8 (4.32–14.09)

ART=antiretroviral therapy; CI=confidence interval; TB=tuberculosis.

**Figure 5:**
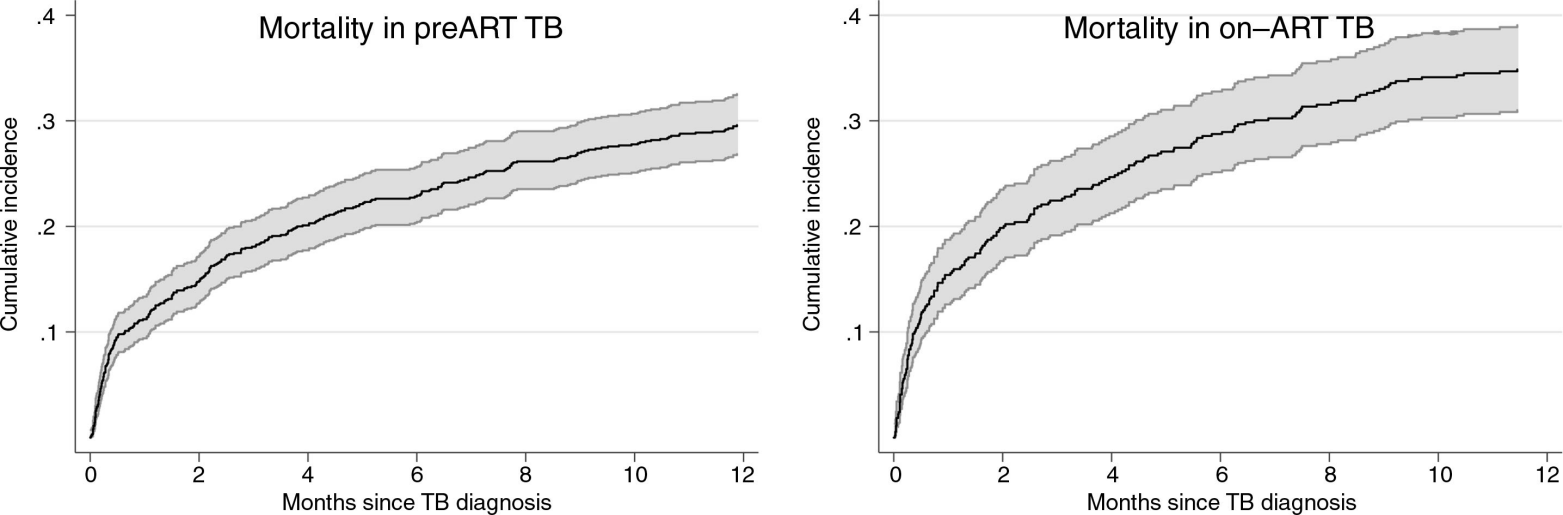
Cumulative incidence of mortality with 95% confidence intervals in patients with tuberculosis (TB) before and after antiretroviral therapy (ART) initiation.

Figure 5 describes the Kaplan-Meier estimates of cumulative incidence of mortality. The cumulative incidence of mortality at 12 months was 29.6% (95% CI, 26.9–32.6) in patients with pre-ART TB and 34.9% (95% CI, 31–39.1) in patients with on-ART TB.

**Figure 6:**
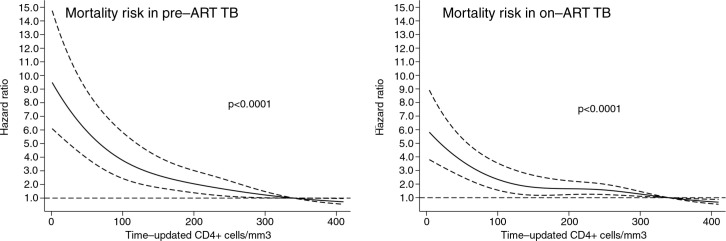
Mortality risk with 95% confidence intervals according to time-updated CD4+ lymphocyte counts of patients with tuberculosis (TB) before and after antiretroviral therapy (ART) initiation.

The relation between mortality and time-updated CD4 lymphocyte counts is presented in Figure 6. While in pre-ART TB the association between low CD4 counts and mortality was approximately linear, this association was mildly weaker in on-ART TB.

**Figure 7:**
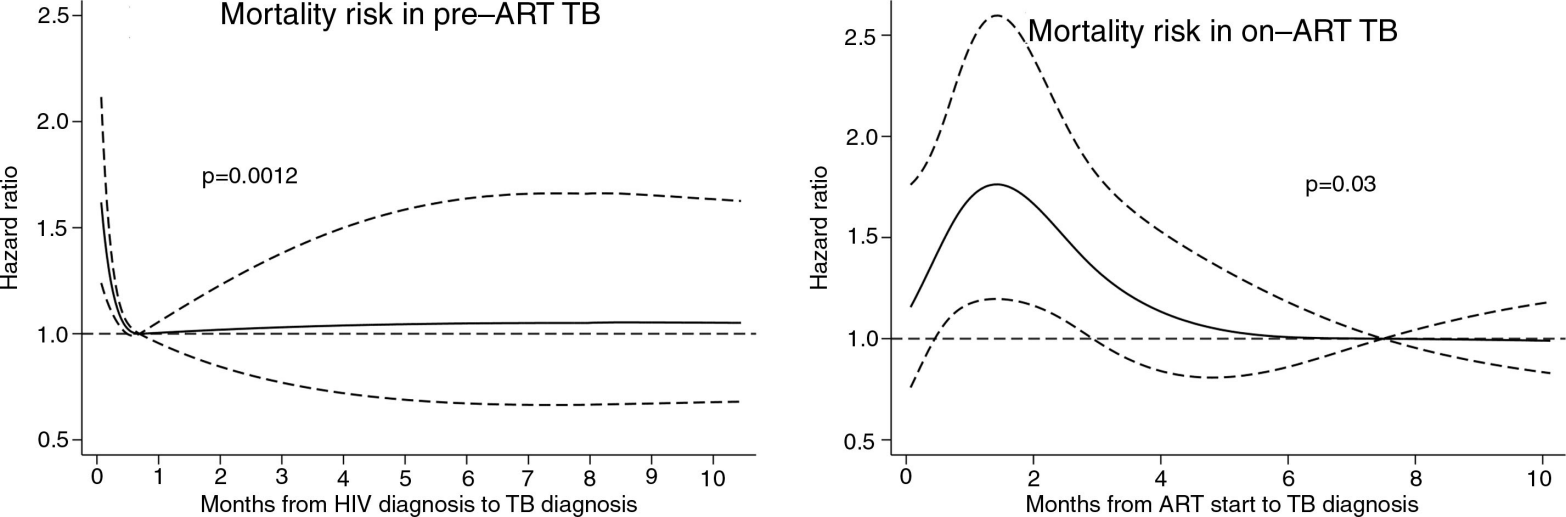
Mortality risk with 95% confidence intervals according to timing of tuberculosis (TB) diagnosis of patients with TB before and after antiretroviral therapy (ART) initiation.

Figure 7 describes the mortality risk according to the timing of TB diagnosis. Patients diagnosed with TB during the first 15 days of HIV diagnosis had about 50% higher risk of death. In on-ART TB, patients diagnosed with TB around six weeks after ART initiation had the highest risk of death, and the mortality risk declined gradually thereafter, reaching a plateau after six months of ART.

### Mortality before and after ART initiation according to TB status

**Figure 8:**
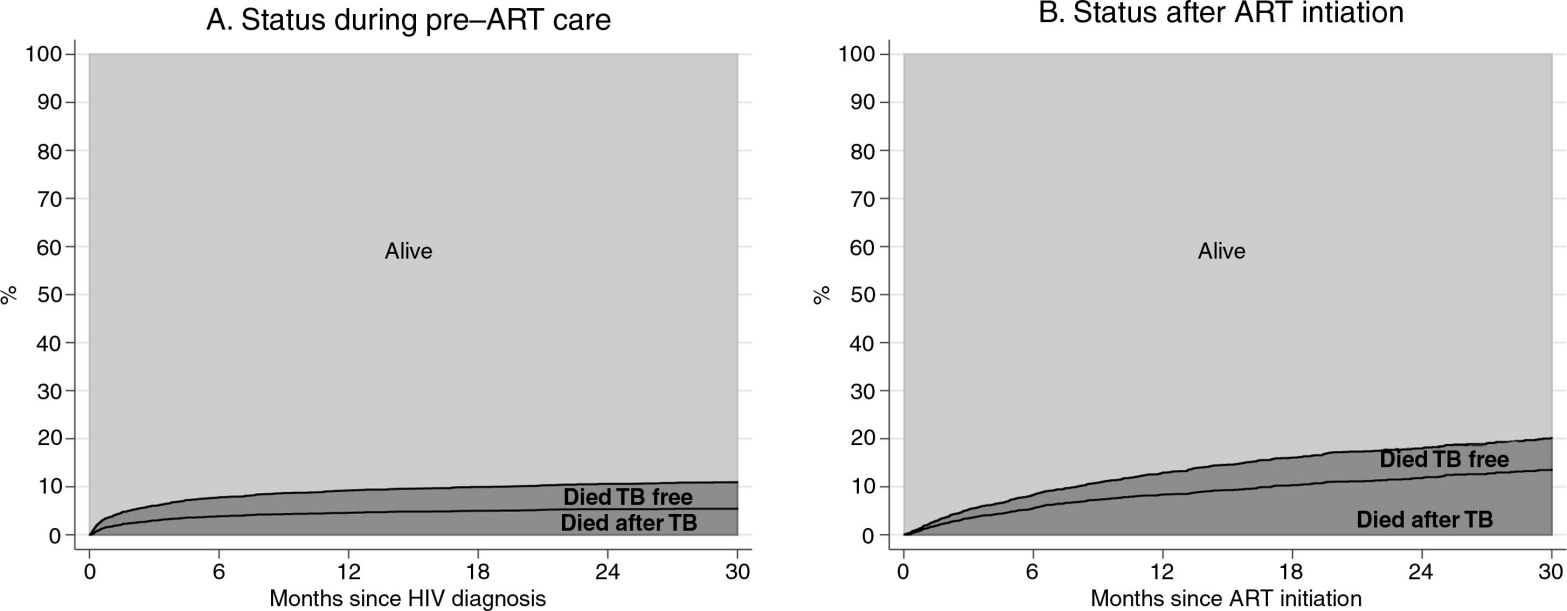
Stacked graphs of the status of patients from HIV diagnosis to antiretroviral therapy (ART) initiation and after ART initiation.

We studied the cumulative incidence of mortality over time from HIV diagnosis to ART initiation and after ART initiation (Figure 8). Patients who died were classified as “death after TB” or “death free of TB” based on whether patients experienced TB before ART initiation or not (Figure 8a) and whether patients on ART received anti-TB therapy or not (Figure 8b). In the pre-ART group, ART initiation was considered a competing event. Thus, deaths after ART initiation were not included in Figure 8a. Consequently, patients who were on anti-TB therapy at the time of ART initiation and died after starting ART were included in the group of “death after TB” in Figure 8b.

The pre-ART mortality at 30 months was 11% (95% CI, 10–12), including 5.47% (95% CI, 4.8–6.2) in patients who experienced pre-ART TB and 5.5% (95% CI, 4.83–6.24) in those not diagnosed with TB (Figure 8a).

The on-ART mortality at 30 months was 20.15% (95% CI, 18.56–21.86), including 13.59% (95% CI, 12.2–15) in patients who experienced TB and 6.56% (95% CI, 5.64–7.56) in those not diagnosed with TB (Figure 8b).

## Discussion

The study describes the epidemiology of TB in a rural district of India with free ART and no isoniazid preventive therapy. Even compared with studies performed in sub-Saharan Africa [29], the incidence rates of TB were extremely high. Our results are in accordance with another Indian study in a different population (the study was originally designed to describe the transmission of HIV in sero-discordant couples), where the incidence rate of TB was 15.4 per 100 py (95% CI, 12.2–19.2) during a median follow-up of 15 months [10]. These staggering figures are not surprising. Forty-six percent of the Indian population have latent TB [30], one of the highest in the world, and over two thirds of HIV-positive patients are diagnosed with HIV when their CD4 counts are <350 cells/mm^3^ [31]. Unfortunately, the current Indian scenario is far from ideal. Isoniazid preventive therapy for HIV-positive patients has not been implemented yet. Infection control is difficult due to overcrowded clinics and that ART centres have been established in space available within existing hospital buildings, which most of times were not designed to reduce the risk of air-borne transmission [32]. Finally, the intensive case finding strategy is far from satisfactory. HIV and TB programmes are not integrated. HIV-positive patients attending HIV diagnosis or ART centres are referred for smear microscopy only when complaining of cough, weight loss, fever or night sweats, despite current evidence showing the suboptimal negative predictive value of this strategy to rule out TB [33,34]. In real life, only 3–5% of patients are referred for smear microscopy [32], and a substantial proportion do not reach smear microscopy centres [35]. Moreover, the sensitivity of smear microscopy to detect TB in HIV-positive patients is poor [34], and 16% of patients found to be smear positive are not linked to anti-TB therapy [32]. The underdiagnosis of HIV-associated TB in India is likely to be overwhelming.

Half deaths before ART initiation and two thirds of deaths after ART initiation occurred in patients with TB. Unlike in developed countries, in India the reduction in HIV incidence has been accompanied by a reduction in the prevalence of HIV, suggesting a high mortality among HIV-positive individuals [36]. Despite similar virological responses to ART [13], in low- and middle-income countries the mortality during the first months of ART is significantly higher than the one in high-income countries [37,38]. The results of our study suggest that this high mortality could be partially explained by the large burden of TB, diagnosed or undiagnosed, in India. Intervention to improve the diagnosis and the prevention of TB could have a major impact in reducing the mortality in HIV-positive patients. In a recent randomized clinical trial, the use of isoniazid preventive therapy reduced the incidence of TB in patients receiving ART irrespective of tuberculin skin test or interferon gamma release assay status [39].

During the first weeks of HIV diagnosis, the incidence rate of TB was extremely high. This may reflect the situation of HIV in our rural resource-poor setting, where two thirds of patients are diagnosed with HIV with CD4 counts <350 cells/mm^3^ [31]. It is likely that many patients with TB were diagnosed with HIV after seeking medical treatment for symptoms secondary to TB. The risk was higher among those with low socio-economical status and low CD4 cell counts. Even after adjusting for other variables, women had lower risk of TB. These findings can be used by HIV programmes to design target-screening interventions aimed at improving the diagnosis of HIV-associated TB.

We found substantial differences between TB before and after ART. Although the incidence rate of TB was higher before ART initiation, patients on ART who experienced TB had higher mortality. In the on-ART group, poor socio-economical conditions were not associated with an increased risk of TB and the association between TB and CD4 counts was less strong and not linear. Our results are in accordance with other studies from low- and middle-income countries. In rural South Africa, incidence rates of TB were three-fold higher in the first three months of ART, especially in those with CD4 lymphocytes count of 50–200 cells/mm^3^, whereas prevalent TB was more frequent in those with CD4 lymphocytes count of <50 cells/mm^3^ [3]. In our study, the risk of TB was highest after two to three months of ART. It is likely that the immune recovery unmasked undiagnosed TB infections present at the time of ART initiation, and these patients had higher CD4 counts and fewer symptoms than those diagnosed with TB before ART initiation. This hypothesis is supported by studies from South Africa showing a high prevalence of smear-negative culture-positive TB in patients starting ART [33,40,41]. These data indicate that we should place more emphasis on screening TB in ART eligible patients before ART is started [42]. However, ruling out TB in this group is challenging because of the poor sensitivity of the sputum smear and the WHO symptom screen [33]. Ideally, sputum culture for mycobacteria should be performed, but it is expensive and difficult to implement in resource-limited settings. Research on new assays suitable for resource-poor settings is desperately needed [43].

The study has some limitations. First, the study was done in a resource-limited setting, so confirmation of TB by mycobacterial culture was not performed. It is possible that some cases were wrongly classified as TB. On the other hand, the lack of mycobacterial culture might have led to an underestimation of the real burden of TB. In anatomical autopsies performed in Africa, over half of HIV-positive people had TB, but the correlation between clinical-determined and postmortem-determined cause of death was poor [44]. In South Africa, TB was found in 87% of early ART deaths [45]. In addition, patients on ART tend to come more frequently to the clinics than patients not on ART [13]. This might have led to ascertainment bias due to greater ascertainment of TB and death in the on-ART group.

## Conclusions

The incidence of TB in our setting was extremely high and the majority of deaths among HIV-positive individuals occurred in patients with TB. We found substantial differences between TB before and after ART initiation, suggesting that future research should consider studying these two entities separately. These findings underscore the urgent need to improve the prevention and diagnosis of HIV-associated TB in India.
